# Annular Basal Cell Carcinoma Expanding Around Central Hypertrophic Scarring: A Case Report

**DOI:** 10.7759/cureus.35934

**Published:** 2023-03-09

**Authors:** Sara Yumeen, Asha Gowda, Mei-Yu Hsu, George Kroumpouzos

**Affiliations:** 1 Dermatology, Warren Alpert Medical School at Brown University, Providence, USA; 2 Dermatopathology, StrataDx, Lexington, USA

**Keywords:** nonmelanoma skin cancer, hypertrophic scar, basal cell neoplasms, basal cell carcinoma diagnosis, fibrosis, scarring, scar, hypertrophic, annular, basal cell carcinoma

## Abstract

A case of annular basal cell carcinoma (BCC) with central atrophic scarring that developed secondary to spontaneous regression has been reported. We present a novel case of a large, expanding nodular and micronodular BCC with annular morphology with central hypertrophic scarring. A 61-year-old woman presented with a two-year history of a mildly itchy lesion on the right breast. Previously diagnosed as an infection, the lesion persisted after treatment with topical antifungal agents and oral antibiotics. Physical examination revealed a 5x6 cm plaque consisting of a pink-red arciform/annular edge with an overlying scale crust and a large, centrally positioned, firm, alabaster-colored portion. A punch biopsy of the pink-red rim revealed nodular and micronodular BCC features. A deep shave biopsy of the central bound-down plaque showed histopathology of scarring fibrosis with no findings of BCC regression. The malignancy was treated with two sessions of radiofrequency destruction, which led to the resolution of the tumor with no recurrence to date.

Contrary to the previously reported case, BCC in our case was expanding, associated with hypertrophic scarring, and showed no signs of regression. We discuss several possible etiologies of the scarring centrally. With further awareness of this presentation, more such tumors can be detected at early stages to facilitate prompt treatment and prevent local morbidity.

## Introduction

Basal cell carcinoma (BCC) shows a wide array of clinical presentations, including pink or pigmented papules/plaques, nodules, non-healing ulcers, scar-like plaques, and pustules. It can cause significant local tissue destruction and morbidity. Our literature review reveals an annular BCC case with central atrophic scarring that developed secondary to spontaneous regression [1]. We present a case of a large, expanding nodular and micronodular BCC with an unusual annular morphology with central hypertrophic scarring. Recognition of this rare presentation is essential to reducing delays in care and associated morbidity.

## Case presentation

**Figure 1:**
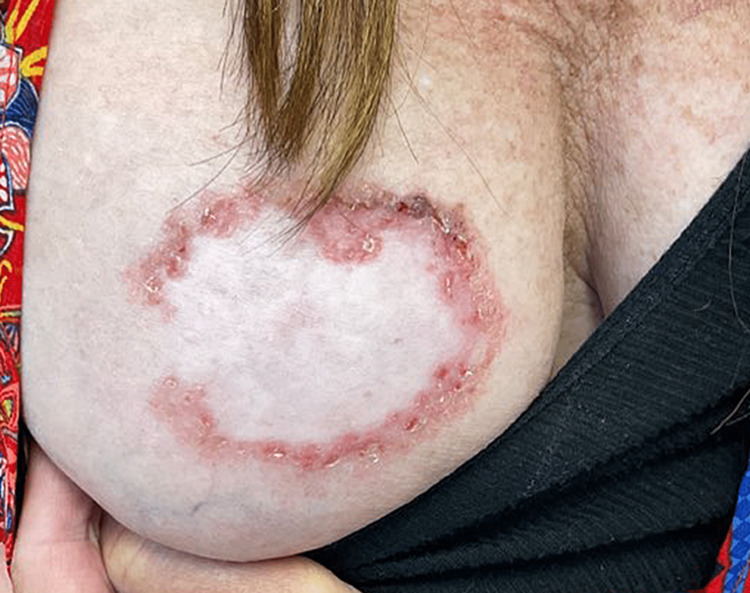
BCC presented as a pink-red arciform/annular plaque with an overlying scale crust. The tumor surrounds firm, alabaster-colored hypertrophic scarring.

**Figure 2:**
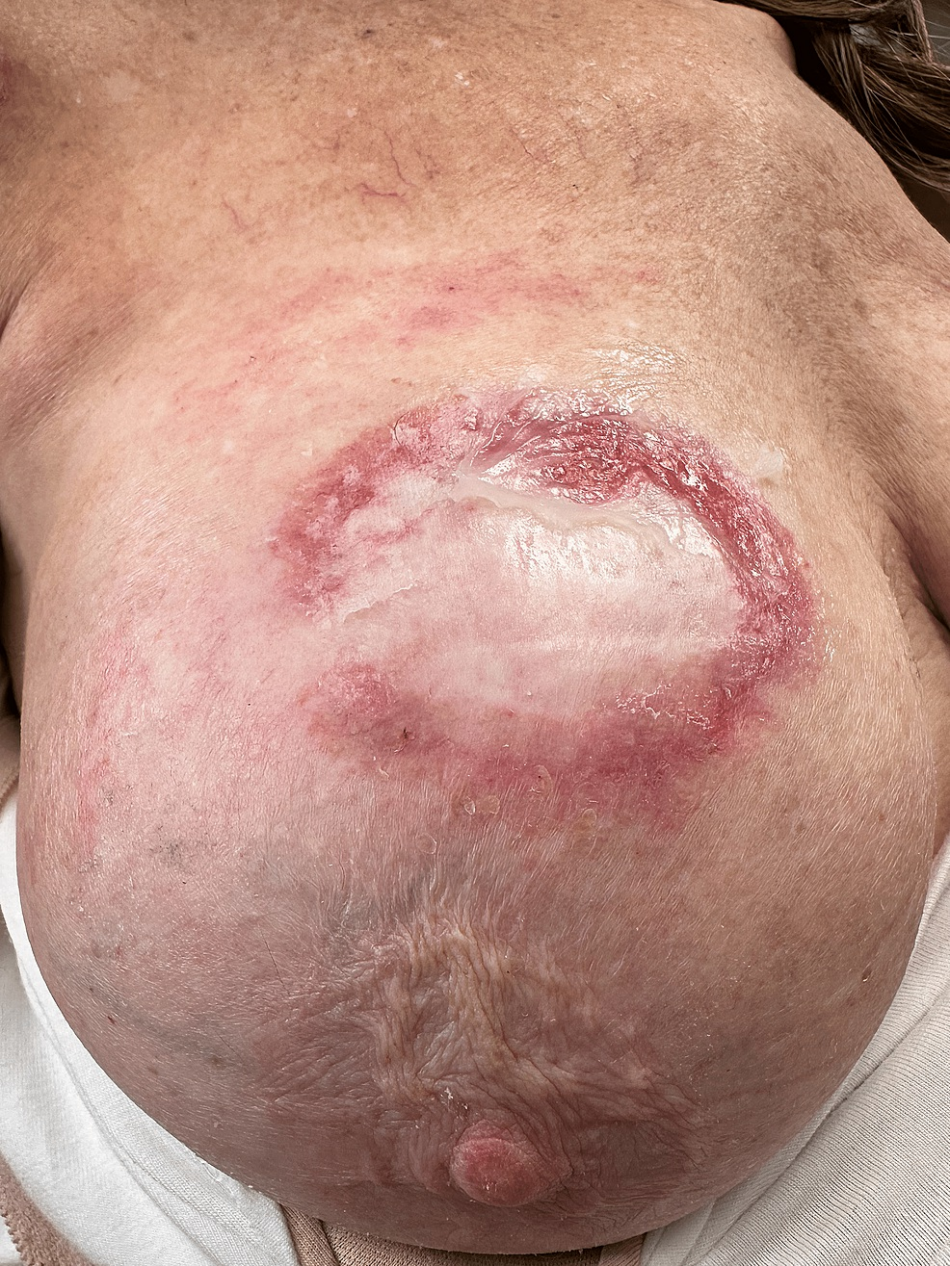
The tumor is shown at the time of radiofrequency destruction. The central scarring is elevated compared to the surrounding normal skin.

A 61-year-old woman presented with a two-year history of a mildly itchy lesion on the right breast. Previously diagnosed as an infection, the lesion persisted after treatment with topical antifungal agents and oral antibiotics. The past medical history included non-melanoma skin cancers and saline-filled breast implantations 30 years prior. Physical examination of the right breast revealed a 5x6 cm plaque consisting of a pink-red arciform/annular edge with an overlying scale crust and a large, centrally positioned, firm, alabaster-colored portion (Figures 1, 2).

**Figure 3:**
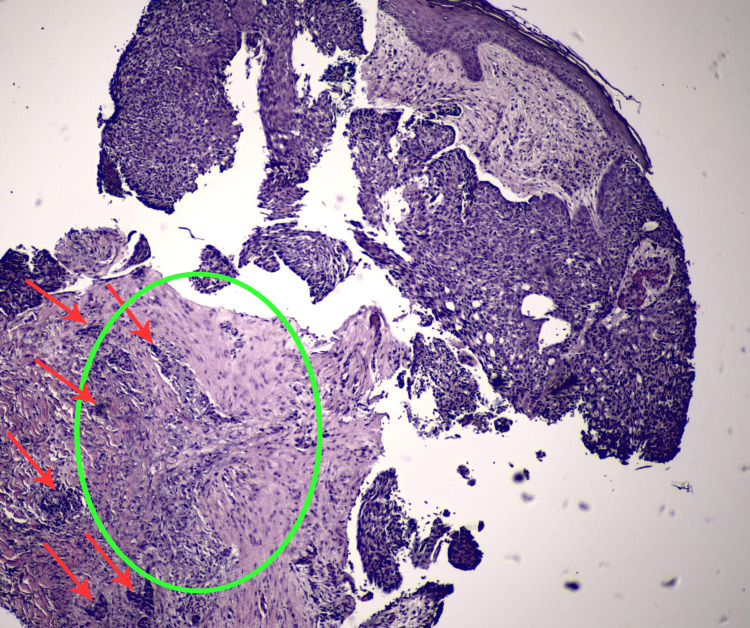
A punch biopsy of the advancing pink-red edge of the right breast plaque shows histopathology of BCC (hematoxylin and eosin stain). Large nodules and small aggregates of atypical basaloid cells (micronodules; shown with red arrows) emanate from the epidermis into the dermis with peripheral nuclear palisading and peritumoral clefting in a fibromyxoid stroma (encircled by the green oval shape) (40x magnification).

**Figure 4:**
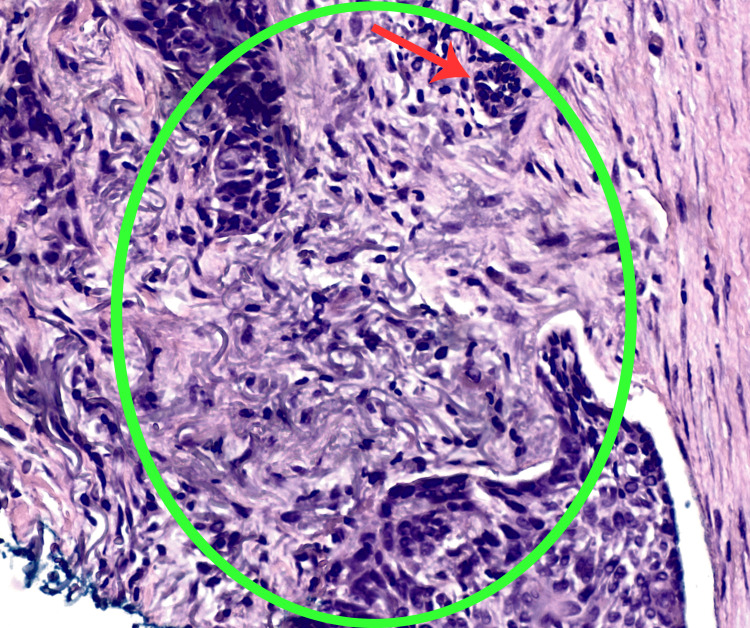
A punch biopsy of the advancing pink-red edge of the right breast plaque shows histopathology of BCC (hematoxylin and eosin stain). Large nodules and a micronodule (shown with red arrow) composed of aggregates of atypical basaloid cells are shown in a fibromyxoid stroma (encircled by the green oval shape) (200x magnification).

There was no cervical, axillary, or inguinal lymphadenopathy. The differential diagnosis included inflammatory skin conditions such as psoriasis, infections such as tinea corporis, morphea, breast tumors such as inflammatory breast cancer, Paget's disease, implant-associated lymphoma, and skin tumors, such as a squamous cell carcinoma (SCC) and BCC, especially the morpheaform type. A punch biopsy of the pink-red rim revealed nodular and micronodular BCC features (Figures 3, 4).

**Figure 5:**
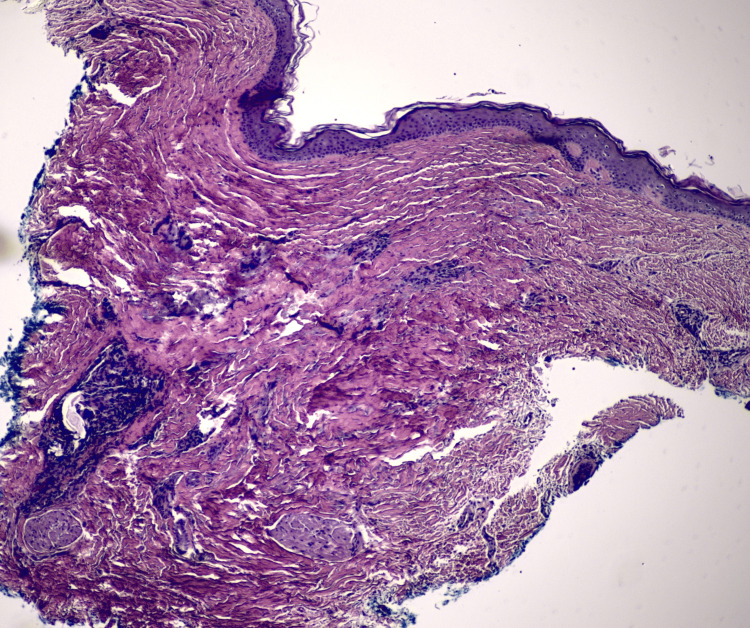
Deep shave biopsy of the central, firm portion of the right breast plaque. Histopathology shows thick collagen bundles in the dermis with patchy lymphohistiocytic inflammation (40x magnification; hematoxylin and eosin stain).

Periodic acid-Schiff-diastase stain was negative for fungi. A deep shave biopsy of the central bound-down plaque showed histopathology of scarring fibrosis without features of BCC (Figure 5).

There were no findings suggestive of BCC regression, such as residuum of the tumor, fibromyxoid stroma typical of BCC, or apoptotic cells, in the biopsy taken from the central scar-like area of the tumor. The tumor lacked typical histopathologic features of morpheaform BCC, i.e., basaloid tumor cells forming infiltrative thin cords in a fibroblast-rich, dense, fibrotic stroma-with the presence of crush artifact and fibromyxoid stroma (Figures 3, 4), a micronodular component is favored. There were no histopathologic features in the central scar-like area, namely, dermal thickening/sclerosis with hyalinization of collagen bundles, hypocellularity, and inflammatory infiltrate with plasma cells at the dermal-subcutaneous junction, to support morphea/scleroderma.

The patient declined surgical excision of the tumor and treatment of central scarring. The malignancy was treated with two sessions of radiofrequency destruction, which led to the resolution of the tumor with no recurrence to date. The patient was relieved of the pruritus and was satisfied with the treatment and healing.

## Discussion

We report a presentation of BCC as an annular expanding plaque with histopathology of BCC at the advancing edge and scarring fibrosis centrally without evidence of tumor. Before the presentation, the tumor had been treated as an inflammatory or infectious condition. Upon questioning, the patient declined that the tumor was initially a solitary pink-red plaque that became annular with central scarring while progressing-she maintained that the lesion had looked annular when she first noticed it. BCCs may grow and cause significant morbidity through local tissue destruction [2]. In our case, the BCC was misdiagnosed because of its atypical presentation, which contributed to a delay in diagnosis and management. As a result, the tumor reached a large size. Delays in care are worrisome for the patient and may result in a significant healthcare burden and require more aggressive treatment.

Of interest, our biopsies demonstrated changes consistent with BCC at the periphery of the plaque, with scar-like changes centrally without evidence of cancer. The scar-like changes centrally may be a result of several possible etiologies. One may consider that, as the BCC’s leading edge progressed, the central portion of the tumor spontaneously regressed with scarring. However, histopathology and our patient's history do not support tumor regression. Also, our case showed central hypertrophic scarring, while previously regressed BCC cases showed central atrophy or depression of the regressing tumor [1,3].

Some authors have hypothesized that central scarring may result from healed ulceration, but our case did not have preceding erosion, ulceration, or crust centrally [3]. Furthermore, while the patient declined a history of trauma to the area prior to lesion development, the possibility of BCC emanating from scar tissue cannot be excluded. While the development of SCC from scar tissue has been more commonly described, BCC arising following trauma has also been reported [4,5]. In cases of BCC arising from dense scar tissue, immunological privilege prevents lymphocyte infiltration, thus interfering with the immune surveillance system [5]. Therefore, the tumor can protect itself from human defense mechanisms until its growth reaches a significant level. Lastly, the fibrosis and dense scar tissue may have been induced by the malignant cells that have transformed to promote scar tissue formation as a mechanism for evading immune detection.

A case of annular BCC with centrifugal spread showed central atrophic scarring without evidence of BCC in the scarred area and reduced cutaneous appendages, including sebaceous and sweat glands and hair follicles [1]. Contrary to our case, the tumor underwent spontaneous regression without intervention. The authors reported the presence of CD8+ and CD68+ inflammatory cells at the periphery of the lesion where BCC was present, suggesting that these cells may play a role in regression. Spontaneous regression has been reported in other cutaneous malignancies, most commonly for malignant melanoma and SCC, keratoacanthoma type. Partial and complete regression has been reported for BCC, with some studies finding up to 20% of BCCs with evidence of at least partial regression [3,6]. Tumor-infiltrating lymphocytes and a type 1 T helper (Th1) cytokine microenvironment have been postulated to play a role in BCC regression [6]. For some cases of complete regression, reports of subsequent recurrence suggest that even regressed areas should be treated for possible residual malignant cells [7].

Curson and Weedon did not report the histological subtype of the regressed BCCs they reported [3]. However, in their cohort of 400 BCCs, they did not find any association between the histological subtype of the tumor and the presence of regression [3]. A case of regressed BCC reported by Go et al. showed both nodular and infiltrative histopathologic features [1]. The currently limited literature suggests that regressed BCC may not be associated with any histopathologic subtype of the tumor.

## Conclusions

Our patient presented with a large, expanding BCC associated with significant hypertrophic scarring. The hypertrophic scarring was not associated with any signs of regression but with the continuous expansion of the tumor. To our knowledge, this presentation has yet to be reported. Possible mechanisms underlying the association between BCC and central scarring are discussed. With further awareness of this presentation, more such tumors can be detected at early stages to facilitate prompt treatment and prevent local morbidity.
